# Complete mitochondrial genome of *Acanthopsyche nigraplaga* (Lepidoptera: Psychidae)

**DOI:** 10.1080/23802359.2021.1899876

**Published:** 2021-03-19

**Authors:** Keon Hee Lee, Min Jee Kim, Ah Rha Wang, Jeong Sun Park, Sung-Soo Kim, Iksoo Kim

**Affiliations:** a Department of Applied Biology, College of Agriculture & Life Sciences, Chonnam National University, Gwangju, Republic of Korea; b Experiment and Analysis Division, Honam Regional Office, Animal and Plant Quarantine Agency, Gunsan, Republic of Korea; c Research Institute for East Asian Environment and Biology, Seoul, Republic of Korea

**Keywords:** *Acanthopsyche nigraplaga*, mitochondrial genome, phylogeny, Psychidae

## Abstract

We report the mitochondrial genome (mitogenome) of a case-making moth *Acanthopsyche nigraplaga* Wileman, 1911 (Lepidoptera: Psychidae). The 15,704 bp long complete mitogenome comprises a typical set of genes [13 protein-coding genes (PCGs), 2 rRNA genes, and 22 tRNA genes] and one major non-coding, A + T-rich region, with an arrangement identical to that observed in most lepidopteran mitogenomes. Twelve of the 13 PCGs of the *A. nigraplaga* mitogenome initiate with a typical ATN start codon, however COI contains the atypical CGA start codon that is common for lepidopteran COI genes. A phylogenetic analysis using concatenated nucleotide sequences of the 13 PCGs and 2 rRNA genes using the Bayesian inference method fully resolved *A. nigraplaga* in a monophyletic clade within the Psychidae. *Acanthopsyche nigraplaga* was situated in a sister position to *Eumeta variegata* and *Mahasena oolona* with high nodal support. As more mitogenome sequences are available further scrutinized analysis for the superfamily Tineoidea including Psychidae will be possible.

*Acanthopsyche nigraplaga* (Psychidae: Lepidoptera) is distributed in Korea, Japan, China, and India (Byun et al. 1996; Roh et al. 2019). Members of the family Psychidae are case-making moths, also called ‘bagworms’, owing to the case-making habit of their larvae. Psychidae is a relatively small family with fewer than 1,350 described species grouped into 241 genera worldwide (Sobczyk 2011), and a limited number of mitochondrial genome sequences have been published. Here, we sequenced the complete mitogenome of *A. nigraplaga* for a subsequent mitogenome-based phylogenetic analysis for the superfamily Tineoidea, in which the family Psychidae is included.

*Acanthopsyche nigraplaga* adults were collected at Bangdong-ri, Sacheon-myeon, Gangneung-si, Gangwon-do, South Korea in 2013 (37°49′18′N, 128°51′59′E). DNA was extracted from their legs and thorax tissue using the Wizard Genomic DNA Purification Kit (Promega, Madison, WI, USA) following the manufacturer’s instructions. The voucher specimen was deposited at the Chonnam National University, Gwangju, Korea, under the accession number CNU13856 (Iksoo Kim, ikkim81@chonnam.ac.kr). Using Lepidoptera-specific primers (Kim et al. 2012), three overlapping long PCR fragments (LFs; COI to ND4, ND5 to lrRNA, and lrRNA to COI) were amplified. These LFs were then used as templates for the amplification of 26 short fragments using Lepidoptera-specific primers (Kim et al. 2012).

The *A. nigraplaga* mitogenome (GenBank acc. no. MT883999) is 15,704 bp in length which is similar to those reported in other Tineoidea including those from Psychidae (Li et al. 2017; Arakawa et al. 2018; Jeong et al. 2018; Roh et al. 2019). The *A. nigraplaga* mitogenome contains a typical set of genes [2 rRNA genes, 22 tRNA genes, and 13 protein-coding genes (PCGs)] and a 258 bp-long major non-coding A + T-rich region. The arrangement of genes in the *A. nigraplaga* mitogenome is the same as that reported in most lepidopteran species (Kim et al. 2010). This arrangement differs from that found in the ancient lepidopteran superfamilies, such as Hepialoidea (Timmermans et al. 2014) and Nepticuloidea (Cao et al. 2012), and from the ancestral arrangement found in the majority of insects (Boore 1999). The A/T content was 77.72% in PCGs, 81.79% in tRNAs, 83.49% in lrRNA, 85.99% in srRNA, and 96.90% in the A + T-rich region. Twelve of the 13 PCGs of *A. nigraplaga* contain ATN as the start codon, except for COI, which initiates with an alternative codon (CGA) as has frequenctly been observed in other lepidopteran insects (Kim et al. 2012).

A phylogenetic analysis was performed using the concatenated nucleotide sequences of 13 PCGs and two rRNA genes (12,368 bp); ten species of Tineoidea, including *A. nigraplaga*, were included in the analysis and represented four families (Psychidae, Tineidae, Meessiidae, and Gracillariidae). *Stigmella roborella*, a species classified in the Nepticuloidea, was utilized as the outgroup. The Bayesian inference (BI) method implemented in CIPRES Portal v. 3.1 (Miller et al. 2010) was conducted using MrBayes ver. 3.2.7 (Ronquist et al. 2012). PartitionFinder2 (Lanfear et al. 2012, 2014, 2016) was used to search for the optimal partitions and the corresponding optimal models of substitution using the ’greedy’ search (GTR + G, TVM + I + G, HKY + G, TVM + G, TRN + G).

The phylogenetic analysis resolved the family Psychidae as a monophyletic taxon with full support (Figure 1). Within the Psychidae, *A. nigraplaga* was inferred as a sister taxon in a basal position to *Eumeta variegata* and *Mahasena oolona*, and *Dahlica ochrostigma* was placed as the most basal lineage in the Psychidae (Figure 1). With respect to familial relationships, the Psychidae and Tineidae formed a sister group with low support [(Bayesian posterior probability (BPP) = 0.58)]. The Meessiidae was placed in a sister relationship to the Psychidae and Tineidae, but support for this relationship was relatively low as well (BPP = 0.55). The Gracillariidae was fully resolved as the most basal lineage of the Tineoidea. Similar phylogenetic results for the familial relationships were obtained by Robinson (1988) and Regier et al. (2013). In contrast, some studies have shown the Psychidae to be the basal group for Tineidae (Mitter et al. 2017; Mutanen et al. 2010; Regier et al. 2015). More complete mitogenome sequences are necessary for investigating the familial relationships of Tineoidea.

**Figure 1.**
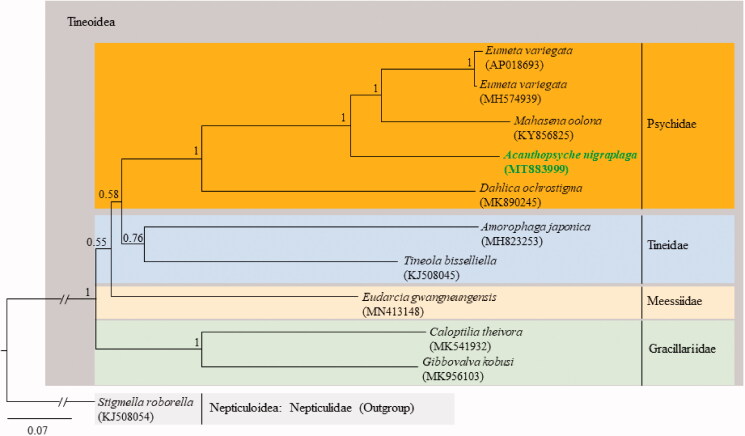
Phylogenetic tree of Tineoidea. The Bayesian inference (BI) method was used for the phylogenetic analysis based on the concatenated nucleotide sequences of 13 PCGs and 2 rRNA genes (12,368 bp) using five partition schemes. The numbers at the nodes indicate the Bayesian posterior probabilities (BPPs) determined using the BI method. The scale bar indicates the number of substitutions per site. The branch length of *Stigmella roborella* was truncated to approximately one-fifth owing to the limited space. *Stigmella roborella* is classified in the Nepticulidae (KJ508054; Timmermans et al. 2014) and was designated as the outgroup. GenBank accession numbers are as follows: *Eumeta variegata*, AP018693 (Arakawa et al. 2018); *Eumeta variegata*, MH574939 (Jeong et al. 2018); *Mahasena oolona*, KY856825 (Li et al. 2017); *Acanthopsyche nigraplaga*, MT883999 (this study); *Dahlica ochrostigma*, MK890245 (Roh et al. 2018); *Amorophaga japonica*, MH823253 (Kim et al. 2020); *Tineola bisselliella*, KJ508045 (Timmermans et al. 2014); *Eudarcia gwangneungensis*, MN413148 (Roh et al. 2020); *Caloptilia theivora*, MK541932 (Chen et al. 2019a); and *Gibbovalva kobusi*, MK956103 (Chen et al. 2019b).

## Data Availability

The genome sequence data that support the findings of this study are openly available in GenBank of NCBI at https://www.ncbi.nlm.nih.gov/nuccore/MT883999.1
